# Sickle Cell Disease Clinical Trials and Phenotypes

**DOI:** 10.4172/2329-891X.1000259

**Published:** 2018-04-08

**Authors:** Chinedu A Ezekekwu, Taiwo R Kotila, Titilola S Akingbola, Guillaume Lettre, Victor R Gordeuk, Richard S Cooper, Michael R DeBaun, Baba Inusa, Bamidele O Tayo, on behalf of the Africa Sickle Cell Research Network

**Affiliations:** 1 Department of Hematology, College of Medicine, University of Ibadan, Ibadan, Nigeria; 2 Montreal Heart Institute, Montréal, QC H1T 1C8, Canada; 3 Division of Hematology & Oncology, Department of Medicine, University of Illinois at Chicago, Chicago, IL, USA; 4 Department of Public Health Sciences, Loyola University Chicago Stritch School of Medicine, Maywood, IL, USA; 5 Guy’s and St. Thomas Foundation Trust, London, United Kingdom; 6 Division of Hematology/Oncology, Department of Pediatrics, Vanderbilt Meharry, Center of Excellence in Sickle Cell Disease, Children’s Hospital at Vanderbilt, Nashville, USA

**Keywords:** Sickle cell disease, Haplotypes, Clinical phenotypes, Malaria

## Abstract

Sickle cell disease, one of the world’s most common genetic disorders is prevalent in sub-Saharan Africa. The trans-Atlantic slave trade accounted for the gene movement from Africa to the Caribbean and United States of America and lately, migration has resulted in the introduction of the gene to the United Kingdom and other parts of Europe. Different haplotypes exist, however the differences in these haplotypes are not sufficient to explain the different clinical variations within the same region or different settings.

## Introduction

Sickle cell disease (SCD), a monogenic disease mostly prevalent in sub-Saharan Africa is one of the world’s most common genetic disorders. The clinical manifestation of which is highly variable despite a more homogenous genotype. The most severe forms seen in sub-Saharan africa and the mildest form in the Indian-Arab haplotype. The phenotype varies within the same haplotype and even among family members. The gene for the disease was initially thought to have been transferred to Africa by nomads from Asia; this arose from the similarities in the blood groups, physical features, agricultural practices and cultural similarities in both populations [1]. More recent evidence however suggests the likelihood of four independent mutations of the sickle cell gene in different populations three of which occurred in Africa [2]. The trans-Atlantic slave trade accounted for the gene movement from Africa to the Caribbean and United States of America and lately migration has resulted in the introduction of the gene to the United Kingdom and other parts of Europe. The incidence of SCD in England (1:200 births) is increasing and the high prevalence is due to the high percentage of births to mothers of West African origin [3,4]. Nearly all white patients with sickling disorders are of Mediterranean origin and carry one of the common African βs-globin haplotype [5]. Studies of homozygous patients with SCD in Saudi Arabia have also shown that patients from the Eastern region have the Asian beta globin haplotype while those from the West have predominantly the Benin haplotype and similar clinical features [6,7]. A combination of DNA markers observed on same chromosome otherwise referred to as haplotypes have been described for SCD. The specific haplotypes found on the chromosomes that carry the HbS variant are named after the geographic regions of Africa and the Middle East where they predominate [8]. These are referred to as Bantu or Central African Republic, Benin, Senegal, Cameroon and Arab-Indian and they vary in the level of fetal hemoglobin and therefore clinical severity. However, the differences in haplotype are not sufficient to account for the clinical variation within the same regions or between different settings. Understanding of these different disease phenotypes deserves urgent attention. For this reason, a multidisciplinary team of experts convened at the Loyola University Medical Center in Chicago, fortuitously, the same city where in 1910, the characteristic sickle red cell was first described by a cardiologist James Herrick [9]. The purpose of the meeting was to identify potential clinical phenotypes that can be studied across low and high-income settings in both children and adults with SCD and amenable to genotypic study. The meeting was also expected to identify potential opportunities for pilot studies.

## Methodology

This multidisciplinary team was made up of research scientists and clinical specialists from the United States of America, Canada, Jamaica, the United Kingdom and Africa (Ghana, Kenya, Nigeria, South Africa and Tanzania). In addition to deliberating on research priorities in children and adults with SCD in both low and high-income countries, it was aimed at creating collaboration among the experts in the fields.

### Prior to the meeting, individuals indicated their area of interest online from the following

- Clinical Trials and Phenotypes
- Birth Cohorts and screening
- Genetics and Molecular

The process began with subgroup discussions weeks in advance of the meeting and this was followed by in-depth debate on identifying the clinical phenotypes to be studied. The following criteria were used to prioritize the phenotypes: generalizable, reproducible, affordable, accessible and clinical relevance.

#### Generalizable

The research area should be applicable to both children and adults and also to both low and high-income countries.

#### Reproducible

The research method should be reproducible and able to consistently give similar results.

#### Affordability

The methods used in carrying out the research should be affordable in large scale and especially in low income countries.

#### Accessibility

The patient groups to be studied and the methods to do so must be easily accessible across the different populations.

#### Clinical Relevance

The research area must be clinically relevant to SCD patients.

An initial list of clinical phenotypes was generated for both adult and paediatric SCD populations (Table 1).

Those generated for the adult group included micro-albuminuria, hemoglobinuria, oxygen saturation, tricuspid regurgitant jet velocity, systemic blood pressure, cerebrovascular disease/silent cerebral infarcts, leg ulcer, pregnancy and perinatal outcomes, priapism, chronic lung disease, forced expiratory volume in the first second, pain and avascular necrosis. The clinical phenotypes in the paediatric age group included pain, alloimmunization, vaccine response, osteomyelitis, transcranial Doppler velocity, asthma, systemic blood pressure, albuminuria, malnutrition as modifier of disease severity, reticulocyte count, gallstones, cognition, splenic function and quality of life.

These were then extensively deliberated upon and a systematic approach by voting was employed in selecting what is most relevant and fits into the established criteria, the list was then trimmed down to four in each group. Later it was agreed upon that malaria chemoprophylaxis which is believed to be relevant to both the adult and paediatric age groups was added. The consensus therefore was to focus on:

1. Albuminuria with assessment of systemic blood pressure, malnutrition as disease severity modifier, vaccine response and abnormal transcranial Doppler velocity in children.
2. Complications of pregnancy and perinatal outcomes, forced expiratory volume in one second, albuminuria with assessment of systemic blood pressure and chronic leg ulcer in adults with SCD.
3. The epidemiology of, immune response to, and optimal chemoprophylaxis for malaria.

## Discussion

The clinical phenotypes of SCD though diverse are age dependent in manifestation. The complications may be broadly classified into hematologic, pain and organ dysfunction [10,11]. These complications arise primarily from either hemolysis or vaso-occlusion and clinical phenotypes of SCD can therefore be ascribed to either or both mechanisms.

Pulmonary hypertension which is thought to be predominantly due to haemolysis is associated with increased mortality [12]. The prevalence is between 6-10% in patients living in high income countries [12], and a study done in a low income country revealed a slightly higher prevalence of 12.2% [13].

A systematic review and meta-analysis reported a prevalence of 20.7% in both children and adult patients [14]. A prospective cohort study revealed that a low forced expiratory volume in one second (FEV1) is associated with early death in SCA [15]. Low FEV1 is typically found in children, adolescent and adults with SCD [15]. Systolic pulmonary artery pressure is easily estimated during echocardiography by the measurement of tricuspid regurgitation velocity (TRV) but elevation of TRV is not always a reliable indicator of pulmonary hypertension [12].

Therefore, diagnosis of pulmonary hypertension may require catheterization after screening is done by echocardiography. However, studies have documented that there is good correlation between TRV measured by echocardiography and systolic pulmonary artery pressure measured by right heart catheterization [16-18]. The problem is that cardiac catheterization may not be readily available in low income settings making TRV by echocardiography the choice in the diagnosis of pulmonary hypertension for studies to be carried out in both low and high-income countries. It may also be useful to combine a study on pulmonary hypertension with that on systemic hypertension since few studies have addressed the latter in SCD patients. The few studies have shown lower systemic blood pressure [19], in SCD, the low systemic blood pressure is believed to be due to a physiological response to the chronic anaemia in these patients.

A relative hypertension, which will include measurements within the higher quartiles of blood pressure for the general population has thus been suggested for SCD patients [20]. Abnormal pulmonary function, most often obstructive is found to be common in children with SCD. It is therefore recommended that full pulmonary function testing be performed in children with SCD, especially when there is a history of asthma or wheezing and accentuated elevations in haemolytic markers [21]. Also, genetic studies have revealed an association between elevated TRV and single nucleotide polymorphisms (SNPs) in GALNTI13 and ADORA2B [22], which are yet to be reported in SCD patients living in Africa.

Renal failure is another cause of early mortality in SCD and occurs in 5-18% of patients [23]. Kidney disease may affect both glomerular and tubular function [24], resulting in proteinuria. The presence of proteinuria appears to be the early sign of renal impairment in high risk patients [22]. About two thirds of adult SCD patients are reported to have detectable proteinuria and over one third in the third decade have albuminuria [24]. For a number of reasons it is thought that hemolysis accounts for renal injury in SCD: HbS as an unstable protein undergoes autooxidation and denaturation to produce oxidants and free heme [25]. There is an inverse relationship between age and estimated glomerular filtration rate (eGFR) in SCD and a low eGFR is found in association with high systolic blood pressure and a high TRV [24]. There is therefore a likelihood of an association between cardiovascular complications and renal disease which may be age related.

It should be noted that creatinine based estimation of GFR results in overestimation of renal function because of tubular hyper filtration of creatinine. In children with SCD, the use of estimated glomerular filtration rate based on creatinine level is not reliable as a result of tubular hyper filtration [19], so tests utilized in assessing renal function should take this into consideration. Though most of the renal manifestations of kidney disease could occur at any age, the sequelae are mostly age dependent. SNPs in the MYH9 and APOLI genes associated with increased risk of chronic kidney disease have been found in association with albuminuria in SCD patients [26,27]. Also, albuminuria correlates with hemolysis, NAG and KIM1 in patients with sickle cell anemia [28]. Conversely, a form of alpha thalassemia has been shown to be protective against albuminuria in these patients [29]. A study of the interactions of the role of these SNPs may become helpful in the pathogenesis of renal dysfunction in SCD.

Maternal mortality from SCD is 14 times higher in low and middle-income countries compared to high income countries and is mostly due to lack of antenatal care [30]. While hypertension, obstetrics haemorrhage and infections account for increased mortality/morbidity, other problems such as cardiovascular diseases remain as important contributors to maternal mortality in both low and high income countries [30]. In view of increased risk of cardiovascular complications, routine screening during pregnancy may be advisable. Prevalent among causes of maternal mortality when SCD patients are compared with controls are fetal anomalies, chronic hypertension, preeclampsia and gestational diabetes [31,32]. Undetected or untreated congenital heart defects, undiagnosed pulmonary hypertension, uncontrolled heart failure are important cardiovascular complications of SCD in pregnancy [30,31]. The odds of preterm delivery and small-for-gestational age are also increased, with 29% of these patients requiring blood transfusion [31,32]. Though there was no significant difference in mortality between transfused and non-transfused patients [32], there is a need to investigate this in a larger cohort of patients.

Chronic leg ulcer as a complication of SCD affects about 8% of sickle cell anaemia patients [33]. Patients who develop leg ulcers are more likely to have severe haemolysis and are therefore more prone to related complications like pulmonary hypertension and priapism [34,35]. Leg ulcers are slow to heal and often recurrent and therefore constitute a huge psychosocial and sometimes financial burden for the patients. A multidisciplinary approach is recommended for its management especially since it affects the quality of life of these patients. There is no reliable therapy for leg ulcer in SCD despite the use of topical and systemic remedies. Similar to chronic leg ulcer, priapism is a debilitating complication of SCD which could occur at any age but has its peak in the 3rd decade [36,37]. The true incidence of priapism is difficult to ascertain because patients with stuttering priapism rarely present to the hospital due to psychological reasons. Another complication of SCD, avascular necrosis (AVN) of the femoral head results from a compromise to the blood supply of the head of the femur. This results in progressive destruction of the femoral head, pain, and ultimate collapse of the hip joint. Treatments for AVN include hip replacement which depends on the extent of damage and patient’s age. Hip replacement is an expensive procedure and may not be affordable for most patients in low-income countries or those without health insurance. AVN has been reported in about 30% of patients [38], and in the most severe form in 8% [33]. Both priapism and avascular necrosis of the femur may be associated with pseudo-addiction to opioids [39], in SCD patients.

Malaria chemoprevention is not uniform across tropical Africa; the practice is common in Nigeria where haemoglobin SS disease (the most severe form of SCD) is prevalent while it is rarely practiced in Ghana where the less severe haemoglobin SC disease is prevalent. Where chemoprophylaxis is practiced both children and adult patients are given prophylaxis though this is hampered by the cost of the medication. There was no difference seen in asymptomatic parasitemia between patients on chemoprophylaxis and those not on chemoprophylaxis, this may be due to the frequency with which patients are treated for malaria even in the absence of positive malaria smears [40,41]. A randomized trial in Nigeria found that intermittent preventive treatment (IPT) with a fixed-dose combination of mefloquine-artesunate was more effective than daily prophylaxis with proguanil [42]. Similar studies in children demonstrate the efficacy of IPT [43,44], in malaria prevention. It may therefore be more effective to apply the intermittent preventive treatment where patients are treated for malaria at intervals rather than placed on chemoprophylaxis; which is now the practice for pregnant and paediatric patients [45]. There is however a need to determine the interval for the treatment and the type of antimalarial drug to be used for SCD patients after appropriate clinical trial. It is also not fully known yet why some individuals die from severe malaria while some individuals remain asymptomatic but there is evidence that 25% of the risk to Plasmodium infection in Africa arise from genetic factors [46]. These genetic differences may likely explain the control of immune responses which partly determine the outcome of the disease [47]. Furthermore, it is known that certain SNPs are protective against uncomplicated malaria and anaemia while others are more susceptible to severe malarial anaemia, cerebral malaria and hyperpyrexia [48]. Most of these studies are done among African SCD patients resident in the continent; comparison with patients in other areas where malaria is not endemic may yield further information.

Vaccination against pneumococcal infection is an effective intervention measure shown to improve survival in SCD patients in developed countries [49]. This is based on the high prevalence of bacterial infection caused by *Streptococcus pneumoniae* in these patients but this high prevalence has not been found in African patients [50]. The differences in the epidemiology of bacterial infection in patients in the tropics will appear to make a case for the non-use of pneumococcal vaccination in the patients. The low rate of occurrence of *Streptococcus pneumoniae* infection in studies done on Nigerian patients may not translate to an absence of the disease but could rather be that patients infected by this organism die before presenting in the hospital or are under diagnosed for other reasons. In many of the Nigerian studies [51-53], on bacterial infections there are not enough patients who are below 30 months of age in contrast to studies in high income countries in which most of the patients are in this age bracket which is the age that the organism is most prevalent [54,55]. A recent study in Kenya showed that Streptococcal infection accounted for 41% of bacterial infection in children with sickle cell anaemia [56]. It may therefore be necessary to vaccinate SCD patients against pneumococcal infection early in life. An understanding of T and B cell lymphocyte function and vaccine reactivity among SCD patients is required for optimization of the use of vaccines in SCD [57]. This is especially important because the disease is associated with elevated levels of inflammatory cytokines and deranged innate immunity, all of which may be associated with diversity in disease presentation and alloimmunization.

Acute painful crisis remains the hallmark of SCD and accounts for 80% of hospital admissions in Britain [3], but only 35% of Nigerian patients [40] and 9.8% of Jamaican patients [58]. These differences may not be due to different genetic or phenotypic type but rather to differing management policies. Painful bone crisis occurs in 70-75% of Nigerian patients in a given year [36,40] and about 25% of these patients report rarely having bone pains, the reasons for this peculiarity is not yet known but is unlikely related to the level of fetal hemoglobin (HbF) [59,60]. Pain is a phenotype that could be studied across low and high income countries and in both pediatric and adult patients. It is also a phenotype that would be of interest to the patient population. It may however be difficult to characterize the different phenotypes for a meaningful genotypic study. Red blood cell alloimmunization will always be a potential complication in SCD as long as blood transfusion is a mainstay of disease management. Altered T cell responses and innate immune abnormalities have been identified in alloimmunized SCD patients [61]. There are reasons to believe that alloimmunization in SCD are associated with chronic pain, end organ damage and shorter survival [62]. Unravelling the basis of these altered interactions at cellular and molecular levels will help the identification of biomarkers that may be beneficial in patient management [60]. Also, examination of such markers across paediatric and adult SCD patients may uncover unrecognized trend in alloimmunization especially its association with pain and organ dysfunction.

Stroke is a cause of irreversible brain damage and with physical and cognitive deficits and may result in death especially in children. It is therefore important to prevent this complication which affects 10% of SCD patients [63]. Transcranial Doppler ultrasonography is useful in identifying patients who are at risk of stroke by measuring flow velocity in large intracranial arteries. Periodic ultrasonography and the selective use of blood transfusion could help in preventing this devastating complication of SCD [64]. Chronic blood transfusion program was found to reduce the prevalence of stoke to 1% [63]. Though, it is recommended that screening for stroke commence at 2 years, less than half of such children undertake the procedure [65]. Once a patient is on the transfusion program, stopping transfusion will reverse the safety of the patient to becoming at risk of stroke [66]. A systematic review showed that the use of hydroxyurea and phlebotomy is not as effective as a transfusion program [66]. Also, more than half of Nigerian parents declined its use in their children [67]. It would therefore appear that chronic blood transfusion remains the mainstay of stroke prevention in SCD children, regardless of its attendant problems of iron overload, alloimmunization and the risk of transfusion reaction and infections.

## Conclusion

Cardiovascular and renal pathologies appear to be areas that could easily be studied in both low and high-income countries in both paediatric and adult patients with SCD, also being an aspect in which the genotypic characters of the phenotype would be an additional benefit. These complications arising from end organ failure may however, not be the priority of SCD patients in whom pain related phenotypes especially those associated with debilitating complications like priapism and avascular necrosis may be of high priority. Pregnancy outcomes and risks associated with pregnancy are of great importance to patients and in addressing the millennium development goals, these could equally be studied across the continents though may not readily be addressed genotypically.

Malaria chemoprophylaxis is only beneficial to areas in which malaria is endemic but it is important to be able to address the question of the benefit of this age long practice and to provide suitable drug(s) if malaria prevention by chemoprophylaxis is still found beneficial.

## Figures and Tables

**Table 1:** Initial list of phenotypes from the SCD Clinical Trial and Phenotypes Group.

**Adult with SCD**	
1.	Microalbuminuria
2.	Hemoglobinuria
3.	Oxygen saturation
4.	Tricuspid regurgitant jet velocity
5.	Systemic blood pressure
6.	Cerebrovascular disease/silent cerebral infarcts
7.	Leg ulcer
8.	Pregnancy and perinatal outcomes
9.	Priapism
10.	Chronic lung disease
11.	Forced expiratory volume in the first second
12.	Pain
13.	Avascular necrosis
**Clinical phenotypes in the pediatric age group**	
1.	Pain
2.	Alloimmunization
3.	Vaccine response
4.	Osteomyelitis
5.	Transcranial Doppler velocity
6.	Asthma
7.	Systemic blood pressure
8.	Albuminuria
9.	Malnutrition as modifier of disease severity
10.	Reticulocyte count
11.	Gall stones
12.	Cognition
13.	Splenic function
14.	Quality of life
